# Design and Rationale of Cytokine Filtration in Lung Transplantation (GLUSorb): Protocol for a Multicenter Clinical Randomized Controlled Trial

**DOI:** 10.2196/52553

**Published:** 2023-12-13

**Authors:** Sandra Lindstedt, Martin Silverborn, Lukas Lannemyr, Leif Pierre, Hillevi Larsson, Edgars Grins, Snejana Hyllen, Goran Dellgren, Jesper Magnusson

**Affiliations:** 1 Skane University Hospital Lund Sweden; 2 Sahlgrenska University Hospital Gothenburg Sweden

**Keywords:** lung transplantation, cytokine filtration, primary graft dysfunction, chronic lung allograft dysfunction, randomized controlled clinical trial

## Abstract

**Background:**

Lung transplantation (LTx) is the only treatment option for end-stage lung disease. Despite improvements, primary graft dysfunction (PGD) remains the leading cause of early mortality and precipitates chronic lung allograft dysfunction, the main factor in late mortality after LTx. PGD develops within the first 72 hours and impairs the oxygenation capacity of the lung, measured as partial pressure of oxygen (PaO_2_)/fraction of inspired oxygen (FiO_2_). Increasing the PaO_2_/FiO_2_ ratio is thus critical and has an impact on survival. There is a general lack of effective treatments for PGD. When a transplanted lung is not accepted by the immune system in the recipient, a systemic inflammatory response starts where cytokines play a critical role in initiating, amplifying, and maintaining the inflammation leading to PGD. Cytokine filtration can remove these cytokines from the circulation, thus reducing inflammation. In a proof-of-concept preclinical porcine model of LTx, cytokine filtration improved oxygenation and decreased PGD. In a feasibility study, we successfully treated patients undergoing LTx with cytokine filtration (ClinicalTrials.gov; NCT05242289).

**Objective:**

The purpose of this clinical trial is to demonstrate the superiority of cytokine filtration in improving LTx outcome, based on its effects on oxygenation ratio, plasma levels of inflammatory markers, PGD incidence and severity, lung function, kidney function, survival, and quality of life compared with standard treatment with no cytokine filtration.

**Methods:**

This study is a Swedish national interventional randomized controlled trial involving 116 patients. Its primary objective is to investigate the potential benefits of cytokine filtration when used in conjunction with LTx. Specifically, this study aims to determine whether the application of cytokine filtration, administered for a duration of 12 hours within the initial 24 hours following a LTx procedure, can lead to improved patient outcomes. This study seeks to assess various aspects of patient recovery and overall health to ascertain the potential positive impact of this intervention on the posttransplantation course.

**Results:**

The process of patient recruitment for this study is scheduled to commence subsequent to a site initiation visit, which was slated to take place on August 28, 2023. The primary outcome measure that will be assessed in this research endeavor is the oxygenation ratio, a metric denoted as the highest PaO_2_/FiO_2_ ratio achieved by patients within a 72-hour timeframe following their LTx procedure.

**Conclusions:**

We propose that cytokine filtration could enhance the overall outcomes of LTx. Our hypothesis suggests potential improvements in LTx outcome and patient care.

**Trial Registration:**

ClinicalTrials.gov NCT05526950; https://www.clinicaltrials.gov/study/NCT05526950

**International Registered Report Identifier (IRRID):**

PRR1-10.2196/52553

## Introduction

Lung transplantation (LTx) remains the standard option for treating patients with irreversible end-stage pulmonary disease [1]. Of all transplanted organs, the mean 5-year survival is the lowest following LTx [2]. The high mortality is highly associated with primary graft dysfunction (PGD), which arises in the first 72 hours after LTx and can lead to chronic lung allograft dysfunction (CLAD) within the first 3-5 years [3]. The cumulative incidence of these 2 conditions is between 40% and 80%. The fear of PGD leads to a low use rate of donated lungs, with only 20% of donated lungs being used [4].

PGD is reflected in the oxygenation capacity of the lung, measured as the ratio of the partial pressure of oxygen (PaO_2_) to the fraction of inspired oxygen (FiO_2_), which decreases with increasing PGD severity [2]. PGD is diagnosed according to International Society for Heart and Lung Transplantation guidelines [5] if the PaO_2_/FiO_2_ ratio drops below 300 and radiographic infiltrates are present in the lungs, consistent with pulmonary edema. Consequently, increasing the PaO_2_/FiO_2_ ratio following LTx is critical and may directly impact survival.

There is a general lack of effective treatments for both PGD and CLAD. When a transplanted lung is not accepted by the immune system in the recipient, a systemic inflammatory response starts where cytokines play a critical role in initiating, amplifying, and maintaining the inflammation leading to PGD [6]. Cytokine filtration can remove these cytokines from the circulation, thus reducing inflammation. Cytokine filtration devices have been shown in vitro and in vivo during orthotopic heart transplantation and kidney transplantation to target and reduce levels of medium– and low–molecular-weight molecules, such as cytokines, pathogen-associated molecular pattern molecules, and damage-associated molecular patterns [7-9]. Other than our feasibility study (ClinicalTrials.gov; NCT05242289) and a case series using extracorporeal cytokine adsorption therapy as rescue after LTx [10], this tool has not been applied to human LTx.

In this study, we want to test whether cytokine filtration improves outcome if administered in conjunction with LTx, for 12 hours in the first 24 hours following the procedure.

## Methods

### Study Design

The SPIRIT (Standard Protocol Items: Recommendations for Interventional Trials) checklist was used when writing this report [11]. This report is based on protocol version GLUSorb-CIP-V1 dated March 4, 2023. All items from the World Health Organization’s trial registration data set are found at ClinicalTrials.gov (NCT05526950).

### Intervention

The CytoSorb Device (CytoSorb) is a single-use, polymer-based adsorption system for the dialysis of physiological fluids in extracorporeal therapies. It consists of a cartridge, containing a cross-linked divinylbenzene polymer that can be connected to various extracorporeal circuits. Blood is pumped through the CytoSorb cartridge and then returned to the body. The beads bind to a wide range of inflammatory mediators, including cytokines and chemokines. The device has been used in several trials, mostly for cardiac surgery patients [12-17]. The device is Conformité Européene–marked and is to be used within its intended use and indication and according to the manufacturer’s instructions.

The CytoSorb device will be connected for 12 consecutive or nonconsecutive hours within the first 24 hours after the end of the surgery. As participants will be in the intensive care unit during this time, adherence to intervention protocol will be the responsibility of the attending physician and perfusionist. The device may be disconnected and reconnected as necessary for the care of the patient, for example during extubation or mobilization. The participant may also be removed from a clinical investigation by the treating physician, and the intervention discontinued if they find that this is in the best interest of the participant. All other concomitant care and interventions will be according to clinical standard procedures for LTx in Sweden.

### Purpose of the Clinical Investigation

The purpose of this clinical trial is to demonstrate the superiority of cytokine filtration in improving LTx outcome, based on its effects on oxygenation ratio, plasma levels of inflammatory markers, PGD incidence and severity, lung function, kidney function, survival, and quality of life (QOL) compared with standard treatment with no cytokine filtration.

### Study Design and Setting

The investigation is a randomized, parallel group, Swedish national multicenter superiority trial comparing cytokine filtration following LTx to standard treatment with no cytokine filtration. A comparator device will not be used in the control group as there is currently no clinically accepted alternative to achieve cytokine removal from the blood, and thus, the control group will only receive standard LTx treatment. The trial sponsor is Region Skåne and SL is the sponsor representative.

### Inclusion Criteria

The participant must meet the following inclusion criterion:

- Eligibility to undergo double LTx at either trial site (Skane University Hospital or Sahlgrenska University Hospital) [18].

### Exclusion Criteria

The participant must not meet any of the following exclusion criteria:

- Age <18 years
- Previous organ transplantation
- Presence of any conditions at the time of surgery that require immunosuppressive therapy. Immunosuppressive therapy is defined as:
  Cyclosporine, tacrolimus, everolimus, or sirolimus—minimum 1 month of treatment prior to transplantation and active treatment at the time of transplantation.
  Any form of antibody-based treatment that is known for having an immunomodulatory effect taken up to 1 week before transplantation.
- Nonconsent

Patients fulfilling the inclusion criterion and none of the exclusion criteria as shown will be randomized to 1 of the 2 groups. Enrollment will continue until the required sample size is achieved or until the interim analysis.

Any substantial modifications to the protocol after this study begins will be duly communicated to them.

### Clinical Investigation Objectives

#### Primary Objective

The primary objective is to determine whether cytokine reduction by cytokine filtration leads to improved oxygenation in patients undergoing LTx.

#### Secondary Objective

The secondary objective is to determine whether cytokine reduction by cytokine filtration improves levels of plasma inflammatory markers (including cytokines), lung infiltrates and oedema, arterial blood gas measures, incidence and severity of PGD, need for norepinephrine, mortality, lung diffusion capacity, spirometry measures, acute rejection episodes, chest computed tomography (CT) findings, CLAD, kidney function, and QOL in patients undergoing LTx.

#### Safety Objective

The safety objective is to determine the number of adverse events and plasma levels of immunosuppressant drugs following cytokine filtration in patients undergoing LTx.

#### Exploratory Objective

The exploratory objective is the collection of lung tissue, blood, bronchoalveolar lavage fluid samples and, when available, explanted tissue from recipients for future exploratory analyses of cytokines and immune cells in patients undergoing LTx with and without cytokine filtration.

### Study Population

This study’s population will consist of a representative group (n=116) of patients with de novo double lung transplants in Sweden who fulﬁll the requirements according to the inclusion and exclusion criteria stated above.

### Outcomes

#### Primary Performance End Point

The primary end point is the oxygenation ratio, defined as the highest PaO_2_/FiO_2_ ratio within 72 hours after LTx.

#### Secondary Performance End Points

The secondary end points are (1) plasma levels of inflammatory markers: interleukin (IL)-10, IL-1β, soluble IL-2 receptor, IL-6, IL-8, and tumor necrosis factor α before and immediately after transplantation and at 6, 12, 24, 48, and 72 hours after transplantation and C-reactive protein and white blood cells before and immediately after transplantation and at day 1, 2, and 3 after transplantation; (2) infiltrates and oedema measured by chest x-ray or CT scan of the thorax at day 1, 2, and 3 after transplantation; (3) highest daily arterial blood gas measurements recorded on day 1, 2, and 3 after transplantation, as a measure of lung function; (4) PGD severity as evaluated per standard clinical procedures using a rating scale from 0 to 3 on day 1, 2, and 3 after transplantation, as a measure of lung function; (5) presence of any grade of PGD on day 1, 2, and 3 after transplantation, as a measure of lung function; (6) total dose of norepinephrine administered in the first 72 hours; (7) mortality up to 48 months after transplantation; (8) lung diffusion capacity, measured by carbon monoxide diffusion, at 6, 12, 24, and 48 months after transplantation, as a measure of lung function; (9) forced expiratory volume, forced vital capacity, and total lung capacity measured by spirometry at 1, 3, 6, 12, 24, and 48 months after transplantation, as a measure of lung function; (10) occurrence of any acute rejection episodes (clinically treated and/or biopsy verified) up to 48 months after transplantation, as a measure of lung function; (11) chest CT findings at 1, 3, 6, 12, 24, 36, and 48 months after transplantation; (12) the presence of any CLAD and CLAD subtypes up to 48 months after transplantation, as a measure of lung function; (13) kidney function evaluated by measurement of serum creatinine levels; calculated glomerular filtration rate and need for renal replacement therapy on day 1, 2, and 3 and at 1, 3, 6, 9, 12, 24, and 48 months after transplantation; urea levels on day 1, 2, and 3 after transplantation; and iohexol-clearance at 3, 6, 9, 12, 24, 36, and 48 months after transplantation; and (14) QOL evaluated by the 36-Item Short Form Health Survey at 24 and 48 months after transplantation.

#### Safety End Points

The safety end points are adverse events, serious adverse events (SAEs), and plasma levels of immunosuppressant drugs (cyclosporine, tacrolimus, everolimus, or sirolimus) in the first 72 hours after transplantation.

#### Exploratory End Points

The exploratory end points are the collection of a portion of routinely collected transplanted lung tissue by transbronchial biopsy and bronchoalveolar lavage fluid (at least at 1, 3, and 12 mo, with additional time points if clinically indicated), collection of explanted recipient lung tissue, and collection of blood samples (at least before transplantation; immediately after transplantation; 6, 12, 24, 48, and 72 h; 1 and 2 wk; and 1, 3, 6, 9, 12, 24, 36, and 48 mo after transplantation, with additional time points if clinically indicated) in a biobank for future exploratory analyses. Sample collection protocols will be detailed in a separate standard operating procedure.

### Methodology

#### Medical Chart Review

The following data will be collected from medical charts at specified timepoints: background variables (demographics, comorbidities, therapies, and perioperative data), FiO_2_ measurements (baseline and 0-72 h after transplantation, adjusted for ventilation), arterial blood gas measurements (baseline and d 1-3 after transplantation), radiological assessments (infiltrates and edema at d 1-3), PGD severity (d 1-3 using a 0-3 scale), total norepinephrine dosage (first 72 h), lung carbon monoxide diffusion capacity (baseline, 6, 12, 24, and 48 mo), spirometry (forced expiratory volume, forced vital capacity, total lung capacity at 1, 3, 6, 12, 24, and 48 mo), chest CT findings (1, 3, 6, 12, 24, 36, and 48 mo), CLAD assessment (presence and subtype up to 48 mo), renal function (serum creatinine, glomerular filtration rate, need for renal replacement therapy at specified intervals), urea levels (d 1-3), iohexol clearance (3, 6, 9, 12, 24, 36, and 48 mo), mortality data (up to 48 mo), acute rejection episodes (occurrence and type up to 48 mo), immunosuppressant levels (first 72 h), adverse events, and SAEs.

#### Follow-Up Questionnaires

QOL will be evaluated at 24 and 48 months after transplantation by designated follow-up assessors who are trained in the administration of the questionnaire. QOL scores will be collected using the Short Form Health Survey questionnaire, which assesses QOL and severity of disease for various chronic conditions [19].

#### Clinical Assessment

Data from assessments and visits will be obtained from the medical records. All data must be supported by the patient’s medical records, that is, source documentation, and subsequently stored in an electronic case record form (eCRF).

#### Safety Assessment

The investigators will report all SAEs and device deficiencies to the sponsor no later than 3 calendar days after they become aware of the event. The sponsor will report to the Swedish Medical Products Agency all of the following reportable events: (1) any SAE that has a causal relationship with the investigational device, the comparator or the investigation procedure, or where such causal relationship is reasonably possible; (2) any device deficiency that might have led to a SAE if appropriate action had not been taken, intervention had not occurred, or circumstances had been less fortunate; and (3) any new findings in relation to any event referred to above. Foreseeable adverse events related to LTx will not be reported [20-26].

#### Data Safety Monitoring Board

Safety reviews and oversight will be under the direction of a Data Safety Monitoring Board (DSMB). The DSMB will be composed of a statistician and 2 physicians (with knowledge in the therapeutic area) who otherwise are not involved in the clinical investigation and are independent from the sponsor, without any competing interests. The DSMB shall be blinded in their safety assessments but can request to unblind the analysis in case of safety concerns. The DSMB will also carry out the interim analysis of the primary outcome variable described in the statistical analysis section. The clinical investigation can be terminated if the DSMB determines that the number of adverse events in the intervention group is unacceptable.

#### Blinding

The physicians, perfusionists, and nurses administering the treatment will not be blinded. Thus, unblinding for these parties is not possible. Outcome assessors for long-term follow-up visits after 1 month will be blinded. Statisticians carrying out the analyses after the end of this study will be blinded. In the case of termination of the clinical investigation, the statisticians will only be unblinded after all analyses are complete, regardless of whether this study is terminated on time or prematurely.

### Statistical Considerations

#### Analysis Population

An intention-to-treat (ITT) analysis population will comprise all patients who have been randomized and who have reached the intervention period (the 24 h immediately after the end of LTx surgery), the so-called modified ITT. Patients who were randomized but did not undergo LTx surgery, who died during surgery, or who did not reach the intervention period for any reason will not be included in this population. Analyses based on this population (modified ITT) will group patients according to the treatment they were randomized to receive, regardless of the actual treatment received. We do not expect missing data for the primary outcome variables. Any missing data will be handled as recommended by Jakobsen et al [27]*.*

#### Descriptive Statistics

Normally distributed continuous data will be presented as the mean (SD) and 95% CIs. Nonnormally distributed continuous data will be presented as the median, IQR, and 95% CIs. Categorical and binary data will be presented as a percentage with CIs, and as absolute numbers. Ordered categorical variables will be presented as the median, IQR, and 95% CIs.

#### Statistical Design, Method, and Analytical Procedures

All statistical analyses will be performed by an independent statistician. Treatment allocation will remain concealed during the initial analysis, with the 2 groups labelled as “group A” and “group B,” ensuring a blinded statistical analysis. All statistical tests will be 2-sided. The distribution of continuous variables will be determined by visual inspection of histograms and quantile-quantile plots. The primary outcome will be based on the mean highest PaO_2_/FiO_2_ ratio within the first 72 hours (a continuous variable). Differences between treatment groups in the mean highest PaO_2_/FiO_2_ ratio within the first 72 hours will be compared using a multivariate linear regression model adjusted for, at minimum, the stratification variables (site, age, and gender). Continuous variables, ordered categorical variables, and binary variables measured over time will be compared using mixed models for repeated measures. The distribution of the data will be checked using quantile-quantile plots, and if the data are skewed, then a generalized linear mixed effects model with the appropriate distribution will be used. A random intercept will be applied at the patient level. We will check model assumptions for each model. Kaplan-Meier survival analysis will be performed for mortality data up to 48 months after transplantation. Median QOL scores from each instrument (ordinal variables) will be compared using the Mann-Whitney *U* test at each time point. Subgroups for analysis shall be described in detail in the statistical analysis plan.

#### Level of Significance and Power

In the interim analysis (described below), the DSMB will use Pocock stopping boundaries (*P*<.03). Therefore, the 2-sided α value in the final analysis will be *P*=.03. We will use a power of 0.8 (80%).

#### Sample Size

The power calculation, performed using GPower 3.1 (AdvanSci Research Solutions), is based on superiority analysis assumptions for the primary end point (improved oxygenation ratio, defined as the highest PaO_2_/FiO_2_ ratio measured within 72 h after LTx). Based on data from a cohort of patients undergoing LTx from Skåne University Hospital in Lund from 2011 to 2020, we estimate that the mean PaO_2_/FiO_2_ value in the control group will be approximately 300 (SD 100) mm Hg and that values will be normally distributed. We estimate that the average PaO_2_/FiO_2_ value will be 60 (SD 100) mm Hg higher (effect size) in the treatment group compared with the control group.

The sample size was calculated using a 2-tailed *t* test to compare the PaO_2_/FiO_2_ values in the treatment versus control groups. We will require 52 participants in each group to achieve the power and α level detailed above. We will add 10% of the required sample size to account for possible dropouts, withdrawal of consent, or mortality in the first 72 hours. Thus, we aim to recruit 58 patients in each group to a total of 116 participants. With this number of participants, we estimate that all secondary outcome variables will have a power of at least 80% at an α level of .05.

#### Expected Dropout Rates

Based on clinical experience, perioperative and postoperative mortality within the first 72 hours after transplantation is expected to be low. The sample size estimation has been adjusted to account for possible losses due to dropouts. Long-term follow-up visits will coincide with standard visits, and thus we do not expect many dropouts during the long-term follow-up.

#### Interim Analysis

The DSMB will perform an interim analysis of the primary outcome after at least 30 participants for each group have undergone LTx and reached the intervention period and the 72-hour time point has been passed for the 30th participant in each group. The DSMB will use Pocock stopping boundaries (*P*<.03) as the statistical limit to guide its recommendations regarding early termination of the trial on the basis of rejecting the null hypothesis. If the *P* value for the primary outcome is below .03 at the interim analysis, then a decision will be taken by the trial management team to either stop the trial early or to continue recruitment. This has been accounted for in the sample size calculation above, and no additional adjustments for multiple testing are necessary.

#### Randomization

An allocation sequence will be created in advance by an independent statistician and linked to a digital randomization module in the eCRF. As each patient is included by the investigator, the next number in the sequence will be revealed and the patient will be assigned to the intervention indicated therein. Randomization is performed using a web-based system (REDCap; Research Electronic Data Capture; Vanderbilt University) and patients are randomized in a 1:1 ratio to 1 of the 2 groups. Randomization will be done using random permuted blocks of varying size, stratified by site, gender, and age.

#### Replacement

Participants who withdraw or are withdrawn from this study or who are lost to follow-up will not be replaced. If a participant’s transplantation surgery is cancelled or rescheduled after they are randomized (they will keep the randomization number if later transplanted), new participants will continue to be randomized until the target number of participants has completed their transplantation surgery.

### Site Monitoring

This study will be monitored by an independent monitor before this study begins, during this study’s conduct, and after this study has been completed, to ensure study conduct in accordance with the clinical investigation plan, accuracy of entries in the eCRF, adherence to the protocol and Good Clinical Practice (GCP), and progress of enrollment. Monitoring standards will be followed and all checks of the consistency of the source data with the eCRFs will be performed according to the monitoring plan.

### Data Management and Data Collection

Forum Söder, a nonprofit organization associated with Lund University and Skåne University Hospital, is contracted for planning and execution of data management activities. Data collected from the patients’ medical records, in paper data logs, and from QOL questionnaires will be transferred into an eCRF in the data capture tool REDCap. Automatic validation programs check for data discrepancies in the eCRFs, and by generating appropriate error messages, allow for modification or verification of the entered data by the investigator staff before being saved in the database.

### Database Management and Quality Control

The monitor will review the eCRFs entered by investigational staff for completeness and accuracy and instruct the site personnel to make any required corrections or additions. Electronic data capture systems will undergo extensive user acceptance testing before they are implemented to ensure that they are verified and validated. To ensure the system is secured, REDCap is located on Windows server 2012 R2 standard, with a firewall. Individual log-in with a 2-factor authentication is maintained. The server is backed-up every night. The sponsor, investigators, statisticians, and trial management team will have access to the final data set.

### Procedures to Protect Participant Confidentiality

REDCap is compliant with GCP standards and fulfils criteria for handling patient data according to Swedish legislation on management of personal data and the General Data Protection Regulation of the European Union. Patients will be pseudonymized through assignment of a study ID number in the database and the code will be safely stored at each site.

### Ethical Considerations

The protocol received approval from Swedish Ethics Committee in September 2022 (registration [daybook] 2022-04567-01) and by the Swedish Medical Products Agency in April 2023 (registration [daybook] 5.1- 2023-23105). Any substantial modifications to the protocol after this study begins will be duly communicated to them. Prior informed consent of the participants regarding participation in the trial and collection of biological specimens will be collected by a trained member of the research team before data collection. All the participants are free to withdraw from this study at any moment without incurring any penalties or consequences with regard to future care or services they might rightfully expect. This study plan has also been reviewed and approved by an independent peer review for funding by the Swedish Research Council (registration [daybook] 2022-00319). Written informed consent will be obtained before any study-related procedures are implemented. This study will be performed in accordance with ethical principles that have their origin in the Declaration of Helsinki and are consistent with ICH-GCP and applicable local regulatory requirements. This study was registered at ClinicalTrial.gov (NCT05526950; September 2, 2022)

### Ancillary and Posttrial Care

At the end of this study, participants will continue to receive whatever care is deemed necessary by their physician, regardless of their assigned intervention.

### Dissemination Policy

The sponsor shall prepare a publicly available clinical investigation report in accordance with regulation (European Union) 2017/745 within 1 year after the end of this study. Planned dissemination of study findings includes paper publication, conference presentations, and community dissemination (eg, community meetings and social network). Professional medical writers may be contracted for preparation of the clinical trial report and publications. There are no plans to make the full protocol, participant-level data set, or statistical code publicly available.

## Results

The recruitment of patients started after a monitor site initiation visit on August 28, 2023. The enrollment is expected to last 3.5 years. The collective rate of LTx at the 2 sites together is expected to be 40-60 patients per year. Since the inclusion criterion is very broad (all patients who are eligible for double LTx in Sweden), we only expect about 1-4 patients per year to be noneligible for this study. Thus, we expect to recruit 30-50 participants per year, making this enrollment time feasible. Long-term follow-up after the end of the investigation is expected to last 4 additional years from the final enrollment. Thus, the investigation and postinvestigation follow-up together are expected to last 7.25 years.

## Discussion

### Overview

Intolerance to a new lung, which leads to PGD, begins already during transplantation and is driven by intense inflammation caused by circulating cytokines [6]. Cytokine filtration can remove the cytokines that lead to intolerance and subsequent PGD from the circulation, thus reducing inflammation, and could therefore improve outcomes in LTx. However, the effectiveness of the CytoSorb device in LTx has yet to be established. Other than our feasibility study (NCT05242289) and a smaller case series that was rescued with extracorporeal cytokine adsorption therapy following LTx [10], this tool has not been applied to human LTx by any clinic within our network or in any trials that are registered at ClinicalTrials.gov or other registries. Therefore, this clinical investigation is justified and relevant as it will establish the effectiveness of cytokine reduction by cytokine filtration using the CytoSorb device in improving lung function following LTx, an area where there is a high unmet need. The clinical investigation is designed to evaluate whether the investigational device is suitable for the purposes and populations for which it is intended within LTx, namely for removal of cytokines in these patients.

Following LTx, the anticipated clinical benefits of cytokine filtration using the CytoSorb device in LTx are reduced severity and incidence of PGD, improved lung function, improved kidney function, improved hemodynamic stability, and reduced mortality.

In a preclinical study of LTx carried out by Ghaidan et al [28], pigs were randomized 1:1 to undergo LTx with or without cytokine filtration with the CytoSorb device during the first postoperative period and followed for 3 days after transplantation. Cytokine filtration significantly improved lung function, evidenced by increased PaO_2_/FiO_2_ ratio, and reduced the incidence of histologically confirmed PGD. Treated animals also had lower levels of inflammatory biomarkers in plasma and bronchial alveolar lavage fluid, lower levels of lymphocytes, neutrophils, and total white blood cells; required less inotropic support; and experienced greater lactate clearance postoperatively.

The mechanisms driving PGD are still not fully comprehended, just as the mechanisms of action behind how the CytoSorb device could improve PGD remain not fully understood. Elevated levels of neutrophil extracellular traps (NETs) have been identified in cases of PGD, underscoring the significance of approaches aimed at mitigating their presence [29,30]. While the precise mechanisms underlying PGD are still not fully elucidated, its association with heightened incidences of both acute and chronic rejection underscores the urgency for the development of proactive interventions [3].

We have recently completed a successful clinical feasibility study of cytokine filtration in conjunction with LTx using a similar protocol as described in this application (ClinicalTrials.gov; NCT05242289). The results from the first 4 randomized patients were published as a brief communication [31]. Treated patients exhibited diminished levels of circulating nucleosomes, as a marker of reduced circulating NETs, and remained free from both PGD and histopathological indications of acute rejection at both 1- and 3-months after transplant. In contrast, patients who did not undergo cytokine adsorption experienced higher levels of circulating nucleosomes, PGD of grades 1 and 3, as well as histopathological signs of acute rejection. Incorporating CytoSorb during transplantation could potentially foster a reduced systemic inflammatory response characterized by lower NET levels, consequently providing support for graft acceptance. We are currently working on finalizing all analyses in the feasibility study. To this point, no adverse events have been reported (unpublished work).

### Limitations

Participant-related factors that may compromise the outcome or interpretation of results include baseline characteristics, demographics, lifestyle, and concomitant medications. Participant-related factors will be mitigated by randomization, exclusion criteria, and stratification by gender and age. Multivariate analyses will be used to adjust for the effect of any differences in demographic characteristics and concomitant medication use between the groups.

Site- and procedure-related factors that may compromise the outcome or interpretation of results include minor local differences in clinical procedures. Site-related factors will be mitigated by stratification of randomization by site to ensure equal numbers of patients are randomized to each intervention at each site. Subgroup analyses will be used to examine the effect of any differences between sites.

### Conclusions

The investigation is a randomized, parallel-group, Swedish national multicenter superiority trial that compares cytokine filtration following LTx to standard treatment without cytokine filtration. This study aims to investigate the effects of cytokine filtration in conjunction with LTx. This treatment is administered for 12 hours within the first 24 hours following LTx with the goal of improving outcomes. It is hypothesized that cytokine filtration will lead to improvements in lung transplant outcome, enhance lung transplant care, and increase overall survival.

## Appendix

Multimedia Appendix 1 Peer-review report by the Swedish Research Council.

## Abbreviations

CLAD: chronic lung allograft dysfunction
CT: computed tomography
DSMB: Data Safety Monitoring Board
eCRF: electronic case record form
FiO2: fraction of inspired oxygen
GCP: Good Clinical Practice
IL: interleukin
ITT: intention-to-treat
LTx: lung transplantation
NET: neutrophil extracellular trap
PaO2: partial pressure of oxygen
PGD: primary graft dysfunction
QOL: quality of life
REDCap: Research Electronic Data Capture
SAE: serious adverse event
SPIRIT: Standard Protocol Items: Recommendations for Interventional Trials

## Data Availability

The data sets generated or analyzed during this study are available from the corresponding author on reasonable request.
