# Supplementary material for: Design and Rationale of Cytokine Filtration in Lung Transplantation (GLUSorb): Protocol for a Multicenter Clinical Randomized Controlled Trial
Source: JMIR Res Protoc. 2023 Dec 13;12:e52553. doi: 10.2196/52553 (PMC10753425; doi:10.2196/52553)
Supplement: Multimedia Appendix 1 [file resprot_v12i1e52553_app1.pdf]

Beredningsgruppens yttrande

2022-00319 Sandra Lindstedt	Beredningsgrupp: KBF-2FA

Utlysningsnamn: Bidrag till kliniska studier inom behandlingsforskning 2022 (Klinisk behandlingsforskning)

Projekttitel (svenska): Cytokinfiltrering vid lungtransplantation - Nationell Svensk Studie (GLUSorb)

Bidragsform: Bidrag till forskningsmiljö

Sökt inriktning: Klinisk behandlingsforskning

Scientific quality of the proposed research 6

1 - Poor, 2 - Weak, 3 - Good, 4 - Very Good, 5 - Very good to excellent, 6 - Excellent, 7 - Outstanding

Open label randomized clinical trial where treatment group will undergo cytokine filtration within 24 hrs after transplantation (LTx) compared with control group (1:1) where no cytokine filtration is performed. Outcome: Primary - Oxygenation ratio, defined as PaO2/FiO2 (P/F) within 72 hrs after LTx. Secondary – QoL, 2-year survival, adverse events, lung and kidney function, incidence and severity of primary graft dysfunction.

Primary graft dysfunction (PGD) after lung transplantation (LTx) remains the leading cause of early mortality and chronic lung graft dysfunction. PGD develops within the first 72 hrs and impairs oxygenation capacity of the lung. There are currently no effective treatments for PGD. Prevention of PGD is crucial and cytokine reduction by filtration has been effective in reducing PGD in heart Tx and in kidney Tx, but not tried in LTx.

The main research questions are motivated and clearly specified. The primary outcomes are well defined and appropriate. The project design is adequate and according to instructions. Variables and measurements, power calculations including drop-outs, sample size and patient selection seem appropriate. The sex and gender issues are relevantly described. Preliminary promising animal data have been published in Nature Communications (2022). Gender issues adequately described.

Patient value – benefit for the society 6

1 - Poor, 2 - Weak, 3 - Good, 4 - Very good, 5 - Very good to excellent, 6 - Excellent, 7 - Outstanding

The results from the study may be directly implementable within few years after completion of study. Targets groups have been consulted including a local questionnaire as well as being involved in planning and evaluation of outcomes (Mer Organ Donation – MOD). Results from the study may contribute significantly to increased clinical benefits and do less harm to the individuals. Further, the study may contribute to better usage of healthcare resources and provide more success full LTx i humans including better usage of the scarce availability of lungs.

Novelty and originality 6

1 - Poor, 2 - Weak, 3 - Good, 4 - Very Good, 5 - Very good to excellent, 6 - Excellent, 7 - Outstanding

It’s a novel approach in LTx and will potentially fill an existing knowledge gap.

Merits of the applicant(s) 6

1 - Poor, 2 - Weak, 3 - Good, 4 - Very Good, 5 - Very good to excellent, 6 - Excellent, 7 - Outstanding

The team has sufficient research experience and scientific network for performing the proposed research. The team has a track record of high quality and ability to disseminate research findings. PI has been involved in critical evaluation of clinical studies or guideline establishment.

Feasibility 3

1 - Not feasible, 2 - Partly feasible, 3 - Feasible

LTx is a highly specialized function in Sweden. The recruitment of 116 patients in toto is feasible within time frame. There is a well described and structured project organization, the team composition and environment are suitable for carrying the proposed research. An experienced core translational research team will be led by PI. The time plan seems reasonable. Collection of data and databases is less well described. Ethical approval has been obtained.

Regions: Only two sites in Sweden are performing LTx (Lund and in Gothenburg) but both sites participate in the current study.

Overall assessment of the application's scientific quality 6

1 - Poor, 2 - Weak, 3 - Good, 4 - Very good, 5 - Very good to excellent, 6 - Excellent, 7 - Outstanding

Strengths:
A well-constructed and well written detailed application. An important research question within scope and a major clinical problem is addressed. It is a novel approach with cytokine filtration for LTx. Randomization is adequately described and clear project organization. Data handling and databases are sufficiently described and a data and safety monitoring board will be established. Variables to support PGD is more clearly described. Statistics appears fine. Good feasibility and merits of the applicant with promising preliminary data which now is published in Nature Communications (2022).

Weaknesses:
None in particular. However, budget seems justified except that bioinformatics is less well described.

Overall, the proposal is of high scientific quality with no significant weaknesses.
